# Supplementary material for: A novel scoring system to predict the requirement for surgical intervention in victims of motor vehicle crashes: Development and validation using independent cohorts
Source: PLoS One. 2019 Dec 10;14(12):e0226282. doi: 10.1371/journal.pone.0226282 (PMC6903719; doi:10.1371/journal.pone.0226282)
Supplement: S1 Table — (DOCX) [file pone.0226282.s001.docx]

**A novel scoring system to predict the requirement for surgical intervention in victims of motor vehicle crashes: Development and validation using independent cohorts**

| S1 Table. Correlations between vital signs at prehospital and upon hospital arrival | | |
| --- | --- | --- |
|  | Correlation* | P value |
| RR | 0.27 | <0.01 |
| HR | 0.67 | <0.01 |
| sBP | 0.57 | <0.01 |
| * = Correlations between prehospital values and those upon hospital arrival. RR = respiratory rate, HR = heart rate, sBP = systolic blood pressure | | |
|  |  |  |
